# Liver-Sparing Total Body Irradiation Using Helical Tomotherapy for Relapsed Pre-B-cell Acute Lymphoblastic Leukemia

**DOI:** 10.7759/cureus.100343

**Published:** 2025-12-29

**Authors:** Romy J Megahed, Gary D Lewis, Somayeh Gholami, April Wurtz, Santanu Samanta, Mausam Patel

**Affiliations:** 1 Radiation Oncology, University of Arkansas for Medical Sciences, Little Rock, USA; 2 Radiation Oncology, The University of Utah, Salt Lake City, USA

**Keywords:** liver, organ sparing, pre-b acute lymphoblastic leukemia (all), radiation oncology, tomotherapy, total body irradiation

## Abstract

Liver toxicity is an uncommon but reported complication of total body irradiation (TBI). The risk of this complication may be magnified in patients with previous hepatic parenchymal disease. In this context, to the best of our knowledge, we present the first case of liver-sparing TBI using helical tomotherapy in a patient with pre-existing liver disease.

In this case, we discuss a 23-year-old male with relapsed precursor B-cell acute lymphoblastic leukemia (pre-B-ALL) who received myeloablative TBI. Tomotherapy allowed us to treat the entire body to 12 Gy in eight fractions, while achieving a mean liver dose of 5.98 Gy, meeting the targeted liver constraint of a mean dose less than 9 Gy. This case demonstrates the feasibility of liver-sparing TBI.

## Introduction

Myeloablative total body irradiation (TBI) is a common radiation therapy practice utilized as part of the conditioning regimen for patients with leukemia undergoing allogeneic hematopoietic cell transplantation (HCT). The purpose of TBI is twofold: eradication of malignant cells and myeloablation (suppression of the immune system to allow engraftment of transplanted stem cells).

Most commonly, an extended source-to-skin distance (SSD) setup is used for efficient and homogeneous dosing, with lung blocks employed to reduce the risk of pulmonary toxicity. However, with the use of helical tomotherapy for TBI delivery, it is possible to spare additional organs [1]. When using tomotherapy for TBI, the lungs and kidneys are typically spared to reduce the risks of pneumonitis and nephrotoxicity, respectively.

The liver is an important organ at risk in TBI, but it is typically not spared. Recent studies suggest it may be an important organ at risk, particularly when the patient is at risk of veno-occlusive disease of the liver (VOD) [2]. VOD is a well-characterized complication of HCT that may worsen with radiation therapy [3,4]. In this study, we report the first case of helical tomotherapy to minimize liver dose during TBI in a patient with hemochromatosis undergoing myeloablative allogeneic HCT.

## Case presentation

A 23-year-old male presented for consultation for the evaluation of TBI as part of the conditioning regimen for HCT in the management of his precursor B-cell acute lymphoblastic leukemia (pre-B-ALL). He was initially diagnosed in 2019 and treated with an induction four-drug regimen according to clinical trial protocol AALL1732, followed by consolidative systemic therapy.

In 2022, he relapsed and was started on mini hyper-fractionated cyclophosphamide, vincristine, doxorubicin (Adriamycin), and dexamethasone (HyperCVD) with inotuzumab. Bone marrow biopsy and flow cytometry showed no residual leukemia. A lumbar puncture (LP) was done, which showed atypical cells. A second cycle of mini HyperCVD with intrathecal cytarabine was given. A repeat bone marrow biopsy and flow cytometry showed no evidence of malignancy.

The patient’s course was complicated by pancytopenia, requiring multiple blood transfusions. He subsequently developed iron overload and secondary hemochromatosis. Allogeneic HCT with a TBI-based myeloablative conditioning regimen was recommended. After multidisciplinary discussion, it was also recommended that the radiation dose to the liver be minimized during TBI. Liver function tests were obtained around the time of radiation therapy, as shown in Table 1, which did not show an increase in liver enzymes.

**Table 1:** Changes in liver function tests at specified time points AST: aspartate aminotransferase; ALT: alanine aminotransferase; LD: lactate dehydrogenase; GGT: gamma-glutamyl transferase; TBI: total body irradiation

Parameter	Two Days Before TBI	One Day After TBI	One Month After TBI
Bilirubin, total (mg/dL) (reference range: 0.1-1.2)	3	2	1.1
				Alkaline phosphatase (U/L) (reference range: 40-130)	44	33	47
AST (U/L) (reference range: 10-40)	31	21	45				
				ALT (U/L) (reference range: 7-56)	50	35	235
LD (U/L) (reference range: 140-280)	178	131	404				
				GGT (U/L) (reference range: 9-48)	35	23	56

Computed tomography (CT) simulation was performed using a closed Aquaplast head-and-shoulder mask (Qfix, Avondale, PA, USA) for head and neck immobilization, with a Vac-Lok cushion (CIVCO Radiotherapy, Coralville, IA, USA) employed to immobilize the pelvis. Four-dimensional computed tomography was not obtained for treatment planning. A slice thickness of 3 mm was used.

A composite tomotherapy plan was created with CTV delineated as the entire body with the liver subtracted. The plan was split into upper and lower body plans, given the height of the patient. The junction of the two plans was placed within the upper legs of the patient. For the tomotherapy plan, a field width of 5 cm was used along with the dynamic jaw mode. The pitch was set to 0.41, and the modulation factor (MF) was set to 2.

**Figure 1:**
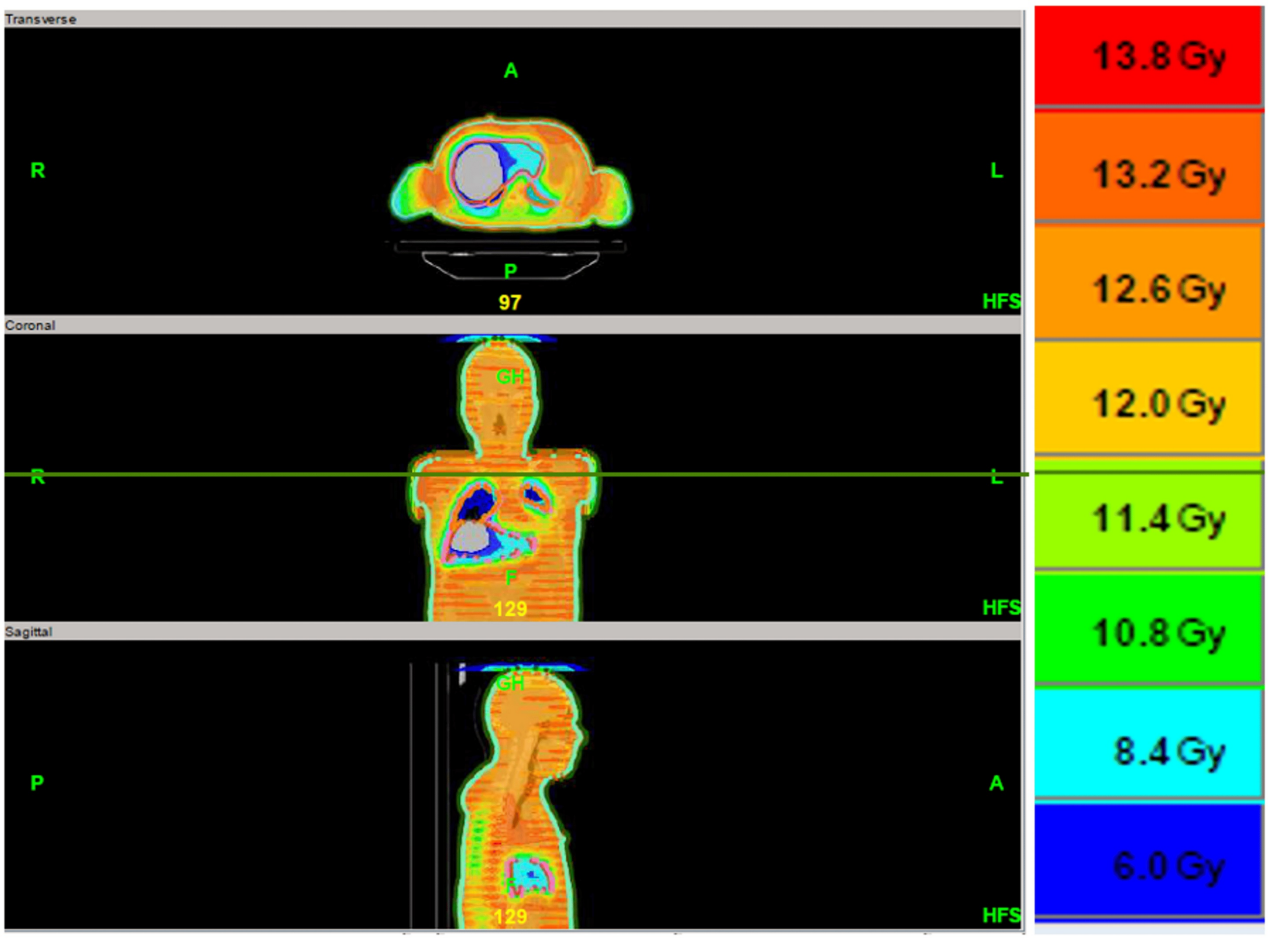
Radiation treatment plan demonstrating liver sparing using helical arc tomotherapy

The radiation treatment plan, as shown in Figure 1, delivered a total dose of 12 Gy in eight fractions administered twice daily, six hours apart, over four consecutive days prior to stem cell transplantation. The liver volume was 1616 cc and received a maximum dose of 11.09 Gy and a minimum dose of 3.91 Gy. The mean liver dose was 5.98 Gy, which was below the targeted constraint of 9 Gy, as shown in Table 2.

**Table 2:** Dosimetry for organs at risk

	Max. Dose (Gy)	Min. Dose (Gy)	Median Dose (Gy)	Avg. Dose (Gy)	Volume (cc)
Liver	11.09	3.91	5.36	5.98	1616
Left lung	13.14	5.47	7.01	7.25	843
Right lung	13.17	4.82	6.26	6.47	1011
Left kidney	11.18	5.23	6.31	6.55	206
Right kidney	11.3	5.37	6.32	6.62	183
Body-liver+5mm	14.1	5.6	12.85	12.62	62408

The patient successfully completed HCT following TBI. Although he required a prolonged hospital stay, he was ultimately discharged home. His post-transplant course was complicated by gastrointestinal graft-versus-host disease and pancytopenia. At 172 days (approximately six months) post-transplant, the patient developed septic shock and was placed on comfort care. There was no evidence of VOD or liver injury.

## Discussion

In this case, we discuss what is, to our knowledge, the first report of liver-sparing TBI in an HCT patient at high risk for liver injury. VOD and radiation-induced liver disease are potentially fatal complications of radiation-based conditioning regimens. Chemotherapy-only regimens do carry variable hepatotoxic risk, particularly with busulfan-containing regimens. While cytotoxic chemotherapy contributes to the development of VOD, it is possible that radiation may exacerbate this. As this was the case, liver-sparing TBI was selected as the treatment of choice. Our patient did not exhibit evidence of VOD or liver injury, although long-term toxicity outcomes were not assessable because the patient died 172 days after the transplant.

Helical tomotherapy was considered in this case based on institutional preference. Consensus guidelines suggest that this approach offers several benefits, including the ability to spare organs at risk as well as treat in a more comfortable supine position [5]. In addition, helical tomotherapy can offer faster treatment times in an HCT patient who may be at high risk for decompensation in an outpatient radiation oncology department.

Organs commonly spared during TBI include the lungs and kidneys [2]. There have also been reports of gonadal sparing in the setting of non-malignant conditions to reduce the risk of azoospermia [6,7]. Total marrow irradiation (TMI) or total marrow and lymphoid irradiation (TMLI) can also be considered to reduce the dose to organs at risk. These techniques selectively target bone marrow or lymphoid tissues rather than the entire body. However, there are no clinical studies that directly compare TMI or TMLI with TBI. Clinical studies examining the use of TMI or TMLI in HCT patients are ongoing.

## Conclusions

TBI remains an important radiation technique that is commonly incorporated into HCT conditioning regimens for the management of malignant and non-malignant hematologic disorders. The ability to deliver uniform dose coverage while simultaneously minimizing toxicity to critical organs is essential to optimize outcomes and reduce morbidity. It is feasible to spare other organs, such as the liver, in cases where patients appear to be at risk for treatment-related toxicity.

In this report, we demonstrate that with the use of helical tomotherapy, liver-sparing TBI is technically feasible and can be achieved with excellent target coverage and a significant reduction in liver dose. Our patient tolerated treatment well, with no evidence of radiation-induced liver injury observed during the follow-up period. Although this represents a single case, these findings highlight the potential role of image-guided, intensity-modulated techniques in improving the therapeutic ratio of TBI. Further investigation in larger patient cohorts will be necessary to establish the efficacy, safety, and long-term outcomes of this approach.
